# Diurnal pattern of salivary cortisol and progression of aortic stiffness: Longitudinal study

**DOI:** 10.1016/j.psyneuen.2021.105372

**Published:** 2021-11

**Authors:** Ai Ikeda, Andrew Steptoe, Martin Shipley, Jessica Abell, Meena Kumari, Takeshi Tanigawa, Hiroyasu Iso, Ian B. Wilkinson, Carmel M. McEniery, Archana Singh-Manoux, Mika Kivimaki, Eric J. Brunner

**Affiliations:** a Department of Epidemiology and Public Health, Institute of Epidemiology and Health, Faculty of Population Health Sciences, University College London, London, UK; b Department of Public Health, Juntendo University Graduate School of Medicine, Tokyo, Japan; c Institute for Social and Economic Research, University of Essex, Essex, UK; d Public Health, Department of Social Medicine, Osaka University Graduate School of Medicine, Osaka, Japan; e Division of Experimental Medicine and Immunotherapeutics, University of Cambridge, UK; f Université de Paris, Inserm U1153, Epidemiology of Ageing & Neurodegenerative diseases, Paris, France

**Keywords:** Cortisol, Aortic pulse wave velocity, Aortic stiffness, Longitudinal study

## Abstract

**Background:**

The positive direct relation between stress and the development of cardiovascular disease has increasingly been recognized. However, the link between hypothalamic-pituitary-adrenal (HPA) dysregulation and subclinical cardiovascular disease has not been studied longitudinally. We investigated the relation of diurnal salivary cortisol, as a biological marker of stress levels, with progression of aortic stiffness over five years.

**Methods:**

A total of 3281 people (mean age 65.5) in the Whitehall II prospective study provided six saliva samples on a single weekday. We assessed the diurnal salivary cortisol using the daytime slope and bedtime level. Aortic stiffness was measured by carotid-femoral pulse wave velocity (PWV) at baseline (2007–2009) and five years later (2012–2013). Linear mixed models were used to estimate the association of diurnal salivary cortisol with baseline PWV and five-year longitudinal changes.

**Results:**

Diurnal salivary cortisol were not associated with PWV at baseline. Among women but not men, a 1-SD shallower salivary cortisol slope at baseline was associated with a five-year increase in PWV (β = 0.199; 95% CI = 0.040, 0.358 m/s) and higher bedtime cortisol level (β = 0.208, 95% CI = 0.062, 0.354 m/s).

**Conclusions:**

Dysregulation of the HPA axis measured using salivary cortisol (shallower slope, higher bedtime level) predicted the rate of progression of aortic stiffness among women.

## Highlights

• The first longitudinal study was conducted to examine the relation of HPA dysregulation with aortic stiffness.
• This study gives some insight into stress-related biological alterations linked to cardiovascular disease development.
• Our findings demonstrated that the effects of HPA dysregulation on the progression of aortic stiffness may be sex-specific.

## 1 Introduction

The positive direct relation between stress and the development of cardiovascular disease has increasingly been recognized in medical literature (Kivimaki and Steptoe, 2018). Plausible explanations include poor adherence to medical regimens and engaging in unhealthy behaviors (e.g. smoking, heavy drinking, being sedentary, poor diet) as well as through potentially toxic direct neuroendocrine effects (Rozanski et al., 2005). Evidence for direct stress-related biological alterations in pathophysiological pathways is sometimes received with skepticism (Black and Garbutt, 2002), however such effects are certainly feasible (Brunner, 1997).

The hypothalamic-pituitary-adrenal (HPA) axis is a major component of the neuroendocrine response to chronic psychological stress. Repeated activation of HPA axis causes HPA axis dysregulation characterized by disturbance of cortisol secretion. Dysregulation of the HPA axis may have adverse effects on the cardiovascular system indirectly and directly; indirectly by inducing hypertension, hypercholesterolemia, or glucose dysregulation, and directly by modifying inflammatory cytokine production and endothelial function (Brunner et al., 2002). HPA axis dysregulation may therefore be important in the development of arteriosclerosis (Hackett et al., 2016, Black and Garbutt, 2002). The hypothesis that HPA axis dysregulation is linked to development of cardiovascular disease implies that ‘stressed’ HPA functioning predicts progression of arteriosclerosis in the general, healthy population (Kirschbaum and Hellhammer, 1989). However, testing this direct stress hypothesis is a challenge and requires a sample of sufficient size to measure the circadian patterns of cortisol and the subsequent age-related changes in a subclinical measure of arteriosclerosis, such as aortic stiffness.

Three lines of evidence lend support to this hypothesis, First, a circadian pattern of cortisol showing a slower and shallower daytime cortisol decline has been linked with higher coronary artery calcification. Reverse causation may be involved as this evidence is cross-sectional (Matthews et al., 2006). Second, both momentary and chronic stress have been linked to a similar change in daytime cortisol pattern (Liao et al., 2013). Third, a shallower daytime cortisol decline predicts incident cardiovascular disease mortality (Kumari et al., 2011) and is linked with other adverse health outcomes (Adam et al., 2017).

We investigated the relation of diurnal salivary cortisol, as a biological marker of stress levels, with the progression of aortic stiffness over five years of follow-up, measured non-invasively by carotid-femoral pulse wave velocity (PWV) (Ben-Shlomo et al., 2014, Cifkova et al., 2019). We hypothesized that participants with a flatter or shallower decline in cortisol through the day at the baseline of the present study would have (i) higher PWV at baseline (2007–2009) and (ii) a greater rate of increase in PWV over time (changes from 2007-2009 to 2012-2013.

The trajectory of change in cardiovascular risk steeply increases in post-menopausal women, potentially at a different rate to the risk in men (Brunner et al., 2011). Moreover, chronic stress associated with traditional gender roles within settings such as the home (e.g., care giving, marital relationship) and workplace (e.g., low autonomy, long working hours) can have a profound impact on cardiovascular health (O'Neil et al., 2018). There is substantial evidence that cortisol responses to stress are greater among women than men (Otte et al., 2005). Thus, we speculated that the association between diurnal cortisol pattern and the progression of aortic stiffness might differ by sex.

## 2 Materials and methods

### 2.1 Study population

**Fig. 1:**
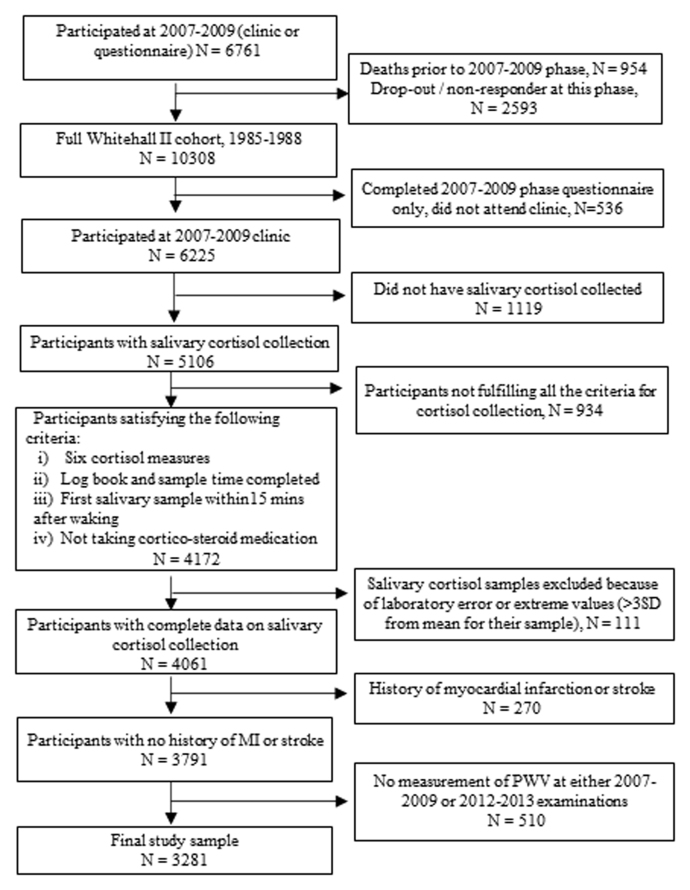
Cohort flowchart.

We used data from the ongoing Whitehall II cohort study. Briefly, 10,308 male and female London-based civil servants, aged 35–55 years, were recruited to the study in 1985, with a response rate of 73% (Marmot et al., 1991). Participants have since been followed up with questionnaire surveys and clinical examinations every 4–5 years. Written informed consent was obtained from all participants at each follow-up clinical examination. The flow diagram indicates the study selection process (Fig. 1). Eligibility for the present study required continued participation from the time when salivary cortisol was collected in 2007–2009 (n = 6225). Of those eligible, 4172 respondents provided six completed cortisol measures, completed the logbook and sample times, were not taking corticosteroid medications, and took their first saliva sample within 15 min of waking based on the protocol for cortisol sample collection. As shown in Fig. 1, 111 participants were excluded due to lab errors or having an extreme cortisol value (i.e., more than three standard deviations (SD) from the mean cortisol concentration for each of their six samples). Participants who have had a past history of myocardial infarction or stroke prior to 2008–2009 were also excluded (n = 270). Further participants who did not have a measurement of PWV at either the 2007–2009 or the 2012–2013 clinical examination were excluded from the final analytic sample. Thus, a total of 3281 participants (men = 2430, women = 851) who had usable cortisol values and at least one measurement of PWV measured during the 2007–2009 (n = 2766) or the 2012–2013 (n = 2798) clinical examination were included in the present study. PWV measures were taken from 70% of participants at both time points (n = 2283). Participant written, informed consent and research ethics approvals are renewed at each contact; the latest approval was by the NHS London—Harrow Research Ethics Committee, reference number 85/0938.

### 2.2 Assessment of PWV

PWV was assessed between the carotid and femoral sites using applanation tonometry (SphygmoCor, AtCor Medical, Sydney, Australia). The time difference between the peak of the R-wave on the electrocardiogram and the foot of the pulse waveform captured by the tonometer at the site of the carotid pulse determined the blood transmission time between the heart and carotid pulse. The blood transmission time between the heart and femoral pulse was measured by the same method. The transit time was defined as the difference between the heart–carotid and heart–femoral blood transmission times. Path length was determined with a tape measure by subtracting the carotid–sternal notch distance from the femoral–sternal notch distance. PWV (m/s) was calculated by dividing the path length by the transit time (Wilkinson et al., 1998). In each participant, PWV was measured twice and if the difference in velocity between the two measurements was larger than 0.5 m/s, a third measurement was taken. The average of the measurements was used in the analysis.

### 2.3 Assessment of diurnal salivary cortisol

The procedure for the collection of salivary cortisol at the 2007–2009 examination has been reported previously (Abell et al., 2016). Participants were requested to provide six saliva samples using salivettes over the course of a normal weekday at waking (sample 1), waking +30 min (sample 2), waking +2.5 h (sample 3), waking +8 h (sample 4), waking +12 h (sample 5), and bedtime (sample 6). Participants were asked to record the time of sample collection in a logbook and were also required to provide information on their wake time. The salivettes and logbook were then returned by post. Salivettes were centrifuged at 3000 rpm for 5 min, resulting in a clear supernatant of low viscosity. Salivary cortisol levels were measured using a commercial immunoassay with chemiluminescence detection (CLIA; IBL-Hamburg, Hamburg, Germany). The limit of detection was 0.44 nmol/l; intra- and inter-assay coefficients of variance were less than 8%. The assay identified any hormone levels over 50 nmol/l needing to be rerun with diluted samples, to check on the validity of the values obtained.

We used five (waking, +2.5 h, +8 h, +12 h, and bedtime) samples to estimate slope in cortisol, with each of the five cortisol values natural log-transformed in order to improve the normality of data distribution (Supplementary Fig. S1). The skewness for each of these five untransformed cortisol measurements were 1.42, 1.81, 1.60, 3.12, and 3.44, respectively. In contrast, the skewness for each of these five log-transformed cortisol measurements were −3.29, −1.50, −1.46, −1.07, and −0.78. Moreover, Q-Q plots of the log-transformed cortisol measurements (Supplementary Fig. S2) suggest adequate normality for the distributions of each of these five log-transformed cortisol measurements as well as the slope in cortisol. As these cortisol samples are clustered within individuals, the slope in cortisol of individuals were estimated using a hierarchical linear model (<Mixed> Stata 14.2; StataCorp, College Station, TX, USA) in which measurement occasion was used as a level 1 identifier and person as a level 2 identifier with sample time as the independent variable and random intercepts and random slopes (Kumari et al., 2011, Abell et al., 2016).

### 2.4 Measurements of other covariates

Every 4–5 years, participants in the Whitehall II study are followed up by clinical examination, a full medical history (myocardial infarction, stroke, hypertension, and diabetes mellitus), and measurement of biomarkers, and complete a questionnaire including lifestyle (smoking, alcohol drinking) and psychosocial (employment grade, depression) factors. Depression was measured using the Center for Epidemiologic Studies Depression Scale (CES-D) (Radloff, 1977). The CES-D consists of 20 items and the sum of all items for each participant provides the total score, which can range between 0 and 60. The British Civil Service employment grade was used as a comprehensive measure of socioeconomic status that reflects education, occupational status, and income, providing a five-level variable (Marmot et al., 1991). Clinical history of myocardial infarction, stroke, hypertension, and diabetes mellitus were ascertained by a self-reported doctor diagnosis on questionnaire surveys. Medication use was examined by questionnaire and the assessment of medications brought by the participant to the clinic visit. Height and weight were recorded in light clothing for the calculation of body mass index (BMI). After a 5 min rest period, systolic blood pressure (SBP) was measured twice using an automated UA-779 digital monitor. Mean arterial pressure (MAP) was calculated as diastolic pressure plus one-third of the pulse pressure. Serum total cholesterol was determined on a Roche P Modular platform after an overnight fast or at least four hours after a fat-free breakfast.

## 3 Statistical analysis

Analysis of variance to compare mean values and chi-square tests to compare proportions were used in the descriptive analyses of the baseline characteristics of men and women. Analysis of covariance was used to compare age-adjusted mean values of baseline PWV (2007–2009) and PWV at 5-year follow-up (2012–2013) between men and women. Linear mixed models were used to measure the effect of diurnal salivary cortisol (per 1-SD increase) on baseline PWV (2007–2009) and PWV longitudinal changes between 2007-2009 and 2012–2013. These models use all available data over the follow-up, handle differences in the length of follow-up, and account for correlation between repeated measures on the same individual. The linear mixed models included a term for time (individual follow-up in years divided by five, to yield effects of change over five years). The main effect estimates the effect of diurnal salivary cortisol on PWV at baseline (2007–2009), whereas the diurnal salivary cortisol × time interaction term estimates the mean difference in the five-year change in PWV. A range of covariates, measured closest in time to when PWV was measured, were included in all multivariable analyses. These were age (years), ethnicity (white or non-white), wake-up time when cortisol was collected (time), employment grade (low-, middle-, or high-grade), CES-D (quartiles), BMI (kg/m^2^), current smoker (yes/no), alcohol intake in the past week (yes/no), total cholesterol (mmol/l), SBP (mmHg), MAP (mmHg), hypertension medication use (yes/no), and history of diabetes mellitus (yes/no). Less than 5% of participants had missing values in one or more covariates. Missing values were replaced by the latest available observation. Although the diurnal slope in cortisol secretion for each participant was estimated using the STATA statistical package version 14.2 (StataCorp), all other analyses were stratified by sex and were conducted using the SAS statistical package version 9.1 (SAS Institute Inc., Cary, NC, USA). Statistical significance was defined as P < 0.05.

## 4 Results

The 3281 participants in the study sample were younger, contained smaller percentages of women and non-whites and generally had a better risk profile than the 2944 participants who attended the 2007–2009 screening clinic but were excluded from the final sample (Supplementary Table S1).

**Fig. 2:**
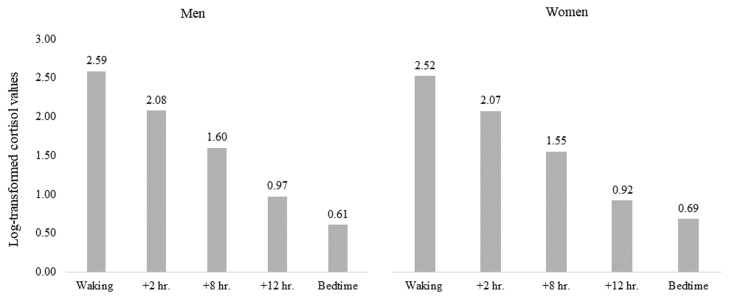
The mean log-transformed cortisol values at each of five points during a day.

The baseline characteristics of men and women are presented in Table 1. The proportions of non-whites, never smokers, non-drinkers, and of a lower grade of employment were higher in women than men. Women were more likely to have higher levels of depression (measured by the CES-D), cholesterol, and bedtime cortisol compared with men. On the other hand, the levels of SBP and MAP were lower in women than in men. The cortisol values decreased by approximately 12% each hour throughout the day (i.e., slope in cortisol) in both men and women (Fig. 2).

**Table 1:** Baseline characteristics (2007–2009) according to sex.

	Men (n = 2430)		Women (n = 851)		
	% or mean	SD	% or mean	SD	P for difference
Age, year	65.5	5.70	65.4	5.77	0.70
Non-white, %	5.32		11.5		<0.001
Low social grade, %	3.21		22.3		<0.001
CES-D^a^	4	1–8	6	2–11	<0.001
Current smokers, %	4.95		3.65		0.12
Alcohol drinkers (in the past week), %	88.3		71.8		<0.001
BMI, (kg/m^2^)	26.2	3.67	26.4	4.98	0.09
Systolic blood pressure, mmHg	125.6	14.9	121.9	16.6	<0.001
Hypertension medication use, %	30.6		30.0		0.72
Arterial pressure, mmHg	90.8	10.4	87.8	11.0	<0.001
Total cholesterol, mmol/l	5.12	1.00	5.58	1.07	<0.001
Diabetes, %	2.22		2.70		0.43
Slope in cortisol, nmol/l^b^	-0.12	0.02	-0.12	0.02	0.47
Bedtime Cortisol, nmol/l^b^	0.61	0.83	0.69	0.84	0.02
Pulse wave velocity, m/s^c^	8.66	2.18	8.44	2.22	0.01

a Shows median and interquartile range and testing the significance performed by Wilcoxon signed-rank test.

b log-transformed.

c earliest measure at either 2007–2009 or 2012–2013.

The age-adjusted mean PWV changed from 8.486 m/s (standard error (SE) = 0.041) at baseline to 9.192 m/s (SE = 0.049) five years later for men and from 8.195 m/s (SE = 0.071) at baseline to 8.928 m/s (SE = 0.085) for women (Supplemental Fig. S3).

The sex-specific multivariable-adjusted mean differences in PWV associated with a 1-SD difference in diurnal salivary cortisol at the baseline (2007–2009) are reported in Table 2. No associations between diurnal salivary cortisol and PWV were evident at baseline in either men or women.

**Table 2:** Association of diurnal cortisol pattern with baseline PWV (2007–2009) and 5-year progression of PWV controlling for demographic, behavioural and biomedical factors.

	Men			Women		
PWV at baseline	Difference^a^	(95%CI)	P-value	Difference^a^	(95%CI)	P-value
Slope in cortisol						
Model 1^b^	0.057	(−0.013,0.127)	0.11	-0.013	(−0.141,0.115)	0.84
Model 2^c^	0.055	(−0.013,0.123)	0.11	-0.030	(−0.158,0.097)	0.64
Bedtime cortisol						
Model 1^b^	0.036	(−0.036,0.107)	0.33	-0.031	(−0.151,0.089)	0.61
Model 2^c^	0.032	(−0.037,0.101)	0.36	-0.056	(−0.177,0.064)	0.36
Change in PWV (per 5 years)	Increase^a^	(95%CI)	P-value	Increase^a^	(95%CI)	P-value
Slope in cortisol						
Model 1^b^	0.043	( −0.055,0.141)	0.39	0.173	(0.013,0.333)	0.03
Model 2^c^	0.034	(−0.064,0.131)	0.50	0.199	(0.040,0.358)	0.01
Bedtime cortisol						
Model 1^b^	0.021	(−0.079,0.120)	0.69	0.181	(0.034,0.328)	0.02
Model 2^c^	0.012	(−0.087,0.111)	0.81	0.208	(0.062,0.354)	0.005

a Differences or increases in PVW are per 1 SD higher value for each diurnal cortisol measure.

b Adjusted for age, ethnic group, wake up time, mean arterial pressure at the time of PWV measurement.

c Further adjusted for social class (low, middle, high), CES-D (quartiles), BMI, systolic blood pressure, hypertensive medication use, total cholesterol, diabetes, alcohol intake, smoking status.

**Fig. 3:**
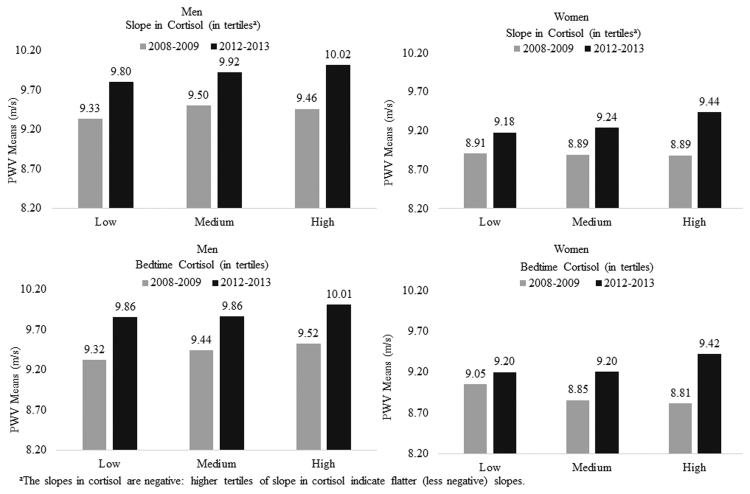
Slope in cortisol (in tertiles^a^) and bedtime cortisol (in tertiles) in relation to PWV by two times points (2008–2009 vs. 2012–2013). ^a^The slopes in cortisol are negative: higher tertiles of slope in cortisol indicate flatter (less negative) slopes.

The sex-specific multivariable-adjusted mean difference in the five-year change in PWV associated with a 1-SD difference in diurnal salivary cortisol is also presented in Table 2. A significant association between the five-year change in PWV and a 1-SD difference in salivary cortisol slope (β = 0.199; 95% confidence interval (CI) = 0.040, 0.358; Cohen’s D = 0.175) and bedtime cortisol (β = 0.208; 95% CI = 0.062, 0.354; Cohen’s D = 0.191) was found in women. Women with flatter slopes of cortisol decline over the day and with higher bedtime cortisol levels at baseline showed larger increases in PWV (Fig. 3). However, no association of diurnal salivary cortisol with change in PWV over time was evident in men.

## 5 Discussion

Our longitudinal analysis found that the rate of increase in PWV as a measure of aortic stiffness over five years was associated with the baseline diurnal pattern of salivary cortisol in women. The greater increase in PWV apparent among women who had flatter daytime cortisol and higher cortisol concentrations at bedtime remained after adjusting for an array of social, behavioral, and biologic factors, suggesting stress-related processes may be involved. We found no association of diurnal salivary cortisol with aortic stiffness in men.

Disturbances in diurnal salivary cortisol have been related to a range of health outcomes apart from coronary heart disease, including diabetes (Hackett et al., 2016), cognitive decline (Gardner et al., 2019), and musculoskeletal strength (Gardner et al., 2013). The present study is the first to provide a direct test of the relationship between diurnal salivary cortisol and changes in PWV, using repeated measures over a period of years. Our findings were consistent with previous research that found an association between diurnal salivary cortisol and cardiovascular mortality (Kumari et al., 2011). Ben-Shlomo et al. (2014) have reported that hazard ratio (95% CI) per SD change in log_e_-transformed PWV was 1.30 (1.18, 1.43) and 1.28 (1.15, 1.43) for the risk of total cardiovascular disease events and cardiovascular mortality, respectively. Combining these results with previous studies may give some insight into the potential functional significance of our findings in relation to the biological factors underlying the association between diurnal salivary cortisol and cardiovascular disease development or progression.

Previous studies have reported mixed results in cross-sectional analyses of diurnal salivary cortisol and subclinical atherosclerosis outcomes. One study found that a flattered diurnal salivary cortisol was associated with increased risk of coronary calcification (CCA) among participants with a mean age of 40 years old (Matthews et al., 2006), while Hajat et al. (2013) reported a null association between diurnal cortisol slope and CCA or ankle-brachial index among participants with a mean age of 65.6 years old. Similar to the findings from Hajat et al., we also did not find a cross-sectional relationship among participants with a mean age of 65.5 years old for men and 65.4 years old for women, but instead found a link between salivary cortisol and trajectories in PWV over time. Matthews et al. (2006) studied a relatively young sample, and it is possible that cortisol is more relevant to the early development of atherosclerosis rather than to people with existing subclinical disease. Thus, the effective impact of diurnal salivary cortisol, in the elderly, may not well reflect overall measures of PWV, but dynamic changes in PWV.

Diurnal cortisol secretion is an important aspect of HPA-axis regulation, in the adaptive responses to chronic stress. An increased cortisol level or a flatter diurnal slope in cortisol may be caused by impairment in the negative feedback of HPA axis activation and the regulation of circadian rhythm as indicative of higher chronic stress (Adam et al., 2017). In the present study, we found that HPA-axis regulation is associated with arterial stiffness in a sex-specific manner. Although the mechanism is not fully understood, sex hormones may be responsible for the sex differences in HPA-axis regulation and may contribute to the increased cortisol response to stress in women (Otte et al., 2005). We also found that bedtime cortisol was higher in women compared to men in the present older adult population. Women tend to be susceptible to chronic stress associated with traditional gender roles in home and workplace (O'Neil et al., 2018), which may have a profound impact on subclinical cardiovascular disease in women. Furthermore, women subjectively experience more stress than men, such that studies of subjective emotion have found that women reported greater sadness and anxiety/fear than men (Fischer et al., 2004). Previous experimental studies have also suggested that there are sex differences in the relationships between stress and behavioral arousal (Zellner et al., 2006) and some markers of cardiovascular risk (Girdler et al., 1990, Rannelli et al., 2017). Besides the sex differences in cortisol stress response, women experience a greater age-dependent increase in arterial stiffness, such that post-menopausal women’s large arteries are stiffer than similarly-aged men (Waddell et al., 2001). The age-adjusted mean PWV change in five years was also higher in women than in men in the present study. These sex differences in biological, social environment, and stress responses may influence the effect of diurnal salivary cortisol on the rate of change in PWV in the present study.

The strengths of the study include the use of a well-characterized cohort of men and women who have been followed over a long period of time. A measure of PWV was obtained twice for most participants (70%). Nevertheless, there are some limitations to the present study. First, most of the associations described have small effect sizes, below 0.2 as indicated by Cohen’s D measure of the standardised effect and confirmatory findings from other studies are necessary before these results can be generalised. Second, the men and women in this study were drawn from the same white-collar occupational cohort and therefore had similar work backgrounds. Thus, further research is needed to assess the possibility that associations between diurnal salivary cortisol and PWV may vary in more diverse populations. Third, we found some evidence for a healthy survivor effect over the follow-up, which most likely led to underestimated effects of diurnal salivary cortisol on aortic stiffness. This might explain why we found associations between diurnal salivary cortisol and longitudinal changes, but no cross-sectional differences in aortic stiffness. Fourth, although cortisol was systematically sampled across the day, measures were taken on only one occasion, so we were not able to evaluate the consistency of assessments or long-term stability. Fifth, although we adjusted for various possible confounding factors in the current study, there is a possibility of residual confounding by unmeasured variables, such as inflammation (Nijm and Jonasson, 2009) and genetic (Kudielka et al., 2009, van den Akker et al., 2008) influences that might account for cortisol response and cardiovascular disease.

In summary, we found that, among women, flatter slopes of cortisol decline over the day and higher bedtime cortisol levels at baseline had greater increases in PWV. The association remained after adjusting for multiple biological and behavioral factors. Our findings also suggest that the pattern of association between the daytime slope and bedtime level of cortisol with cardiovascular health outcomes may be sex-specific.

## Source of funding

Whitehall II study is supported by grants from British Heart Foundation, UK (RG/16/11/32334), UK Medical Research Council, UK (S011676, R024227), and the US National Institute on Aging, USA (R01AG062553, R01AG034454). Ikeda is supported by Japan Society for the Promotion of Science, Japan (JP17KK0175). Brunner is supported by British Heart Foundation, UK (RG/16/11/32334). Kivimäki is supported by NordForsk, the Nordic Programme on the Health and Welfare, Academy of Finland, Finland (311492) and Helsinki Institute of Life Science, Finland. McEniery is supported by the National Institute for Health Research Cambridge Biomedical Research Centre, UK. Funding sources did not have a role in the design or conduct of the study; the collection, management, analysis, or interpretation of the data; or the preparation, review, approval, or decision to submit the manuscript.

## Declaration of Competing Interest

The authors have nothing to disclose.
