# Supplementary material for: Diurnal pattern of salivary cortisol and progression of aortic stiffness: Longitudinal study
Source: Psychoneuroendocrinology. 2021 Nov;133:105372. doi: 10.1016/j.psyneuen.2021.105372 (PMC8543075; doi:10.1016/j.psyneuen.2021.105372)
Supplement: Supplementary file 1 — Supplementary material [file mmc1.docx]

Table S1: Comparison of characteristics of 6225 participants who attended the screening clinic in 2007-2009, according to whether they are in the final study sample

| . | In final study sample | | | | |  |
| --- | --- | --- | --- | --- | --- | --- |
|  | Excluded  (N= 2944) | |  | Included  (N=3281) | |  |
|  | % or  mean | SD |  | % or  mean | SD | P for  difference |
| Age, year | 66.3 | 6.12 |  | 65.5 | 5.72 | <0.001 |
| Female, % | 31.6 |  |  | 25.9 |  | <0.001 |
| Non-white, % | 8.9 |  |  | 6.9 |  | 0.004 |
| Low social grade, % | 12.7 |  |  | 8.2 |  | <0.001 |
| CES-D ^a^ | 6 | 2-12 |  | 4 | 1-9 | <0.001 |
| Current smokers, % | 6.8 |  |  | 4.6 |  | <0.001 |
| Alcohol drinkers (in the past week), % | 77.6 |  |  | 83.9 |  | <0.001 |
| BMI, (kg/m^2^) | 27.5 | 4.9 |  | 26.2 | 4.1 | <0.001 |
| Systolic blood pressure, mmHg | 126.7 | 17.1 |  | 124.6 | 15.4 | <0.001 |
| Hypertension medication use, % | 43.2 |  |  | 30.5 |  | <0.001 |
| Arterial pressure, mmHg | 89.7 | 10.8 |  | 89.7 | 10.5 | 0.92 |
| Total cholesterol, mmol/l | 5.22 | 1.16 |  | 5.24 | 1.04 | 0.32 |
| Diabetes, % | 4.8 |  |  | 2.4 |  | <0.001 |

^a^ Shows median and interquartile range with test of difference performed by Wilcoxon signed-rank test.
