# Supplementary figures and images for: Diurnal pattern of salivary cortisol and progression of aortic stiffness: Longitudinal study
Source: Psychoneuroendocrinology. 2021 Nov;133:105372. doi: 10.1016/j.psyneuen.2021.105372 (PMC8543075; doi:10.1016/j.psyneuen.2021.105372)

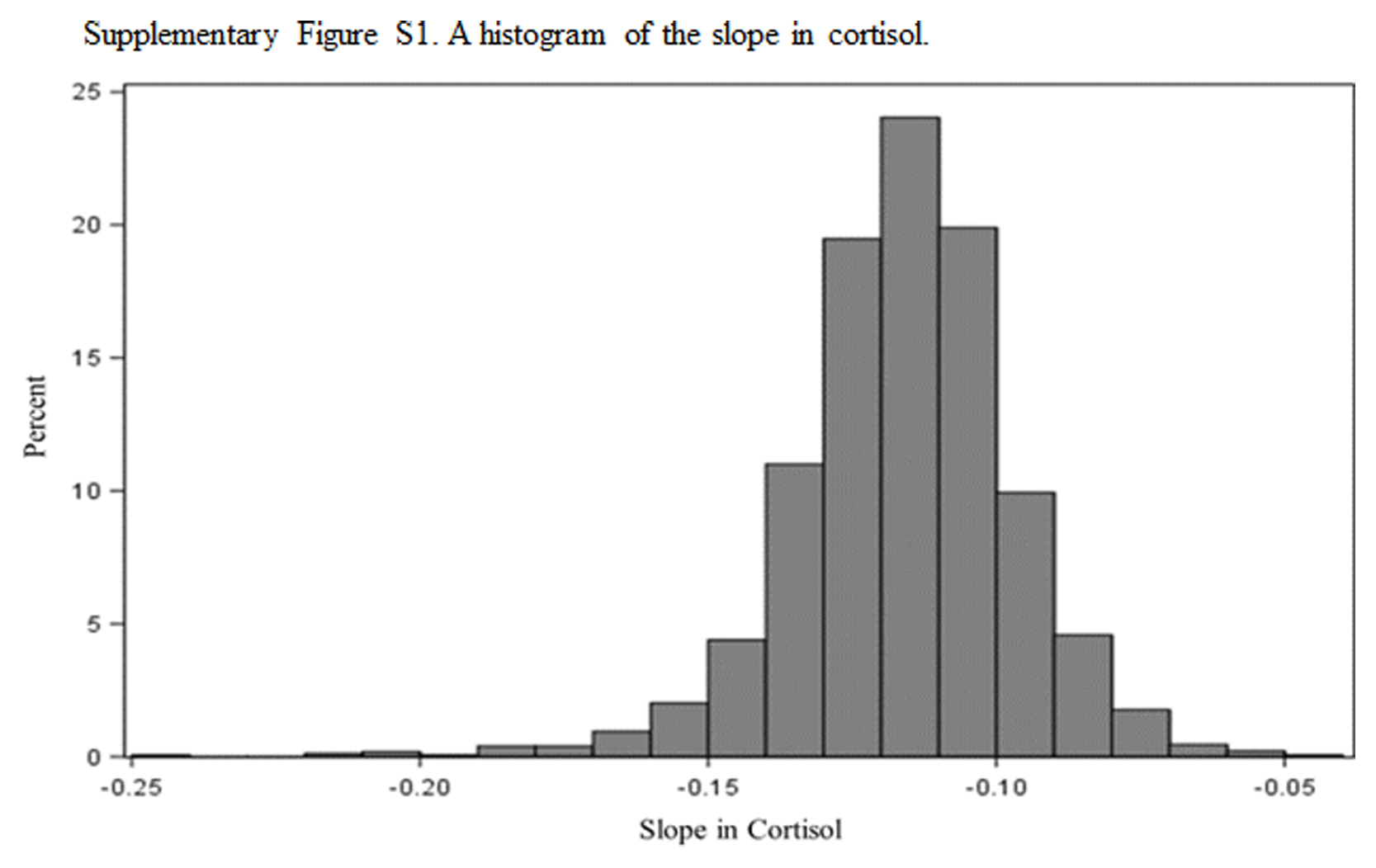

Supplement: Supplementary file 2 — Supplementary material [file mmc2.jpg]

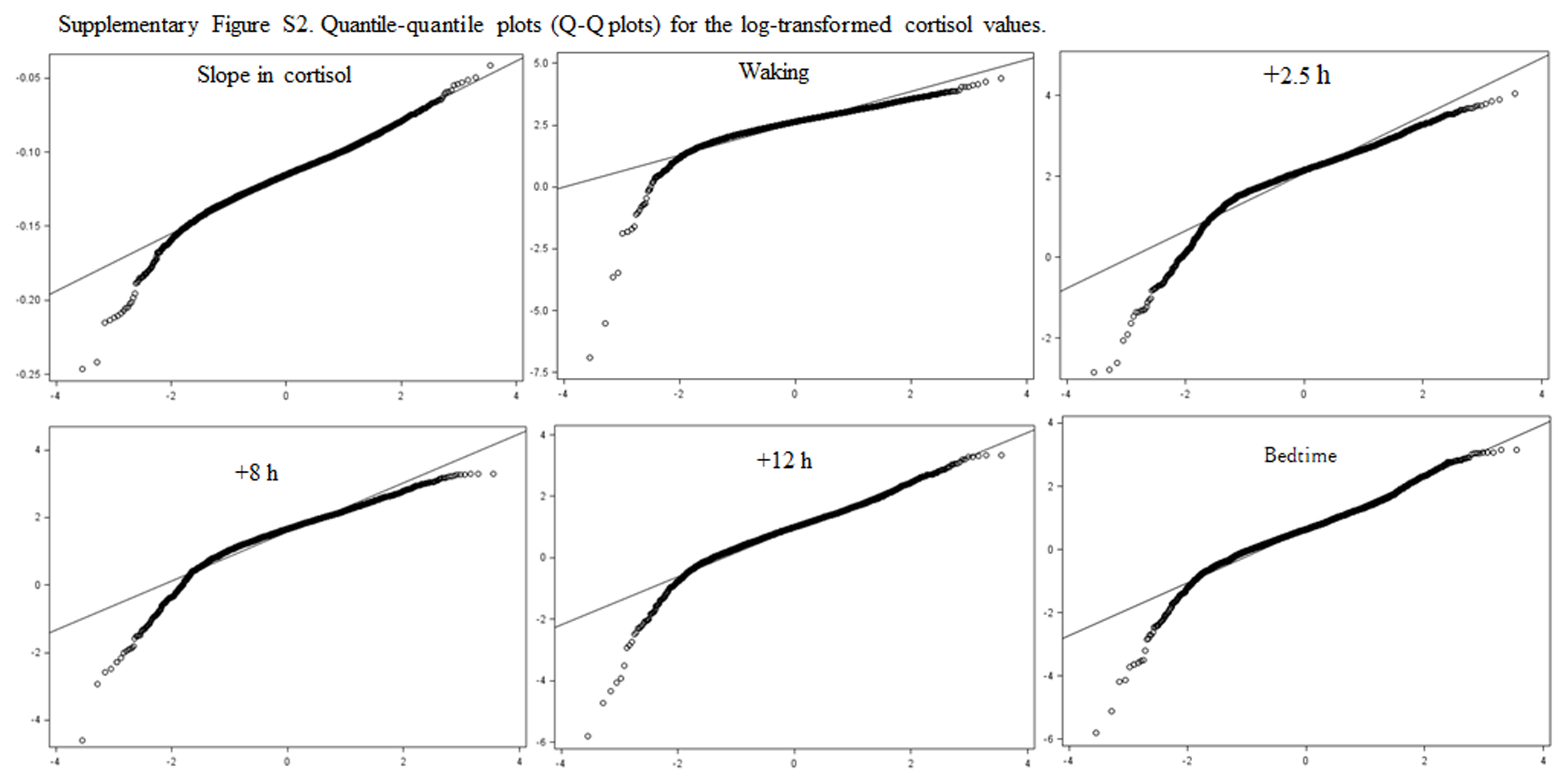

Supplement: Supplementary file 3 — Supplementary material [file mmc3.jpg]

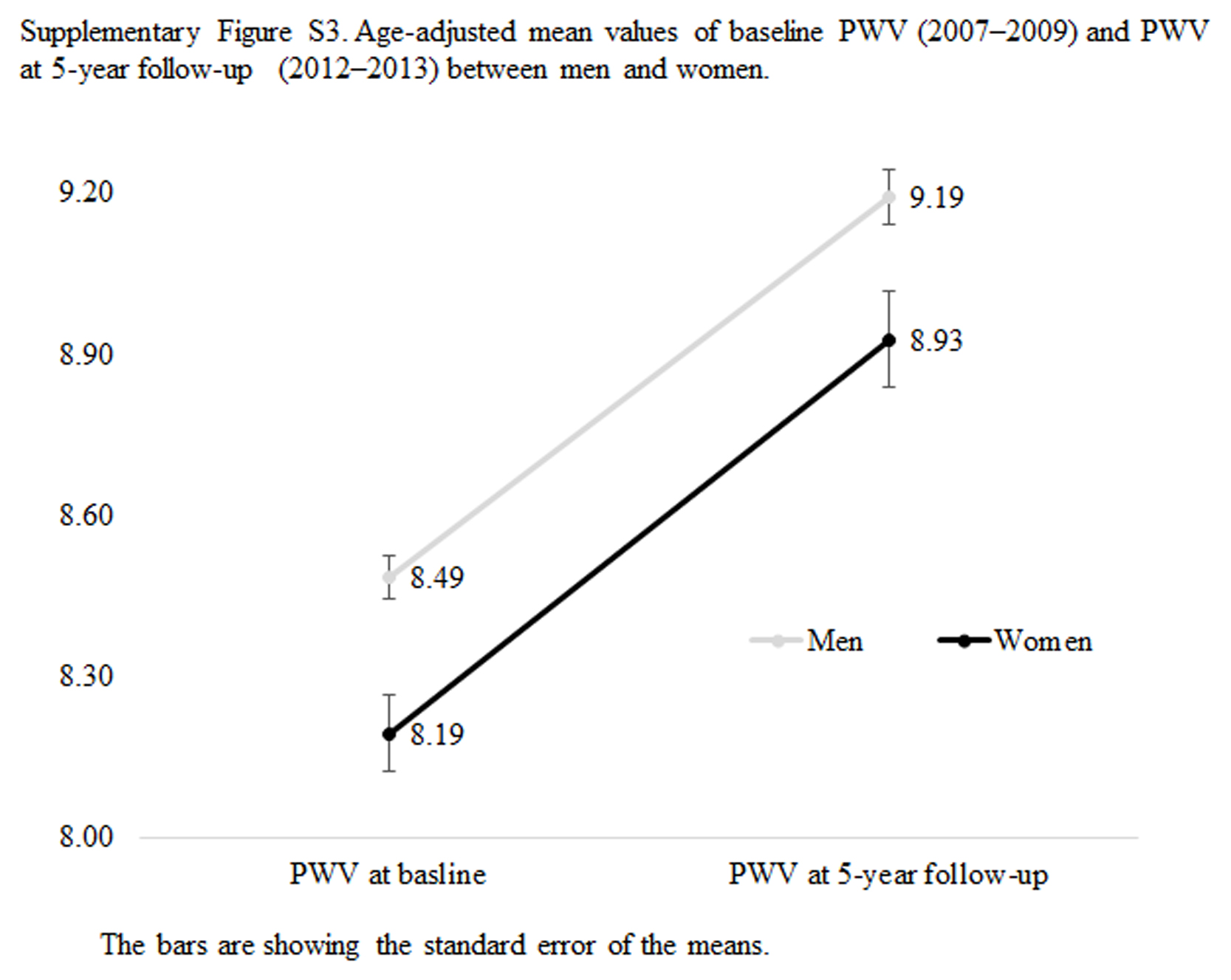

Supplement: Supplementary file 4 — Supplementary material [file mmc4.jpg]
